# Mapping and Validation of Quantitative Trait Loci on Yield-Related Traits Using Bi-Parental Recombinant Inbred Lines and Reciprocal Single-Segment Substitution Lines in Rice (*Oryza sativa* L.)

**DOI:** 10.3390/plants14010043

**Published:** 2024-12-26

**Authors:** Ghulam Ali Manzoor, Changbin Yin, Luyan Zhang, Jiankang Wang

**Affiliations:** 1 State Key Laboratory of Crop Gene Resources and Breeding, Institute of Crop Sciences, Chinese Academy of Agricultural Sciences, Beijing 100081, China; alibaloch1878@gmail.com (G.A.M.); zhangluyan@caas.cn (L.Z.); 2 State Key Laboratory of Plant Cell and Chromosome Engineering, Institute of Genetics and Developmental Biology, Innovative Academy of Seed Design, Chinese Academy of Sciences, Beijing 100101, China; yinchangbin@genetics.ac.cn

**Keywords:** yield-related traits, QTL mapping, QTL validation, recombinant inbred lines (RILs), single-segment substitution lines (SSSLs), rice (*Oryza sativa* L.)

## Abstract

Yield-related traits have higher heritability and lower genotype-by-environment interaction, making them more suitable for genetic studies in comparison with the yield per se. Different populations have been developed and employed in QTL mapping; however, the use of reciprocal SSSLs is limited. In this study, three kinds of bi-parental populations were used to investigate the stable and novel QTLs on six yield-related traits, i.e., plant height (PH), heading date (HD), thousand-grain weight (TGW), effective tiller number (ETN), number of spikelets per panicle (NSP), and seed set percentage (SS). Two parental lines, i.e., *japonica* Asominori and *indica* IR24, their recombinant inbred lines (RILs), and reciprocal single-segment substitution lines (SSSLs), i.e., AIS and IAS, were genotyped by SSR markers and phenotyped in four environments with two replications. Broad-sense heritability of the six traits ranged from 0.67 to 0.94, indicating their suitability for QTL mapping. In the RIL population, 18 stable QTLs were identified for the six traits, 4 for PH, 6 for HD, 5 for TGW, and 1 each for ETN, NSP, and SS. Eight of them were validated by the AIS and IAS populations. The results indicated that the allele from IR24 increased PH, and the alternative allele from Asominori reduced PH at *qPH3-1*. AIS18, AIS19, and AIS20 were identified to be the donor parents which can be used to increase PH in *japonica* rice; on the other hand, IAS14 and IAS15 can be used to reduce PH in *indica* rice. The allele from IR24 delayed HD, and the alternative allele reduced HD at *qHD3-1*. AIS14 and AIS15 were identified to be the donor parents which can be used to delay HD in *japonica* rice; IAS13 and IAS14 can be used to reduce HD in *indica* rice. Reciprocal SSSLs not only are the ideal genetic materials for QTL validation, but also provide the opportunity for fine mapping and gene cloning of the validated QTLs.

## 1. Introduction

Rice (*Oryza sativa* L.) is a staple food crop for approximately 50% of the world’s population. Similar to many other major crops, the enhancement of grain yield has always been the primary objective of rice breeding [1,2]. Understanding the molecular and genetic bases of breeding-targeted traits can help to design the most efficient crossing and selection methods, and therefore to improve the breeding efficiency [3,4,5]. As the most important targeted trait of breeding, grain yield is also the ultimate trait, which can be affected by many factors during the entire growing period of rice plants. As a result, grain yield is probably the most complicated trait in genetics and quantitative genetics; i.e., heritability at the individual plant level is low, the number of affecting genes is large, effects of individual genes are small, environmental and random error effects can be great, and both inter-genic epistasis and genotype-by-environment interactions may occur [6]. These characteristics make it more challenging to investigate its genetic architecture, when compared with a number of related traits. Plant height (PH), heading date (HD), thousand-grain weight (TGW), effective tiller number (ETN), number of spikelets per panicle (NSP), and seed set percentage (SS) are some of the most important examples of yield-related traits.

Generally speaking, yield-related traits are less complicated in genetics and have been more frequently used in quantitative trait locus (QTL) mapping, fine mapping, and map-based cloning. PH is highly related to plant architecture, biomass, and lodging in rice. In addition to the most famous semi-dwarf gene of green revolution, i.e., *sd1* [7], a number of QTLs with relatively smaller genetic effects have been identified on PH, such as *qPH7-2*, *qPH6-3*, *qph1-2*, *qph9-1*, *qPH-1a*, *qPH-1b*, *qPH-2b*, *qPH-3b*, *qPH-6*, and *qPH-7b* [8,9,10]. HD is a key factor that is related to rice adaptation, cropping seasons, maturity, and grain yield. As examples, *Hd-1*, *Hd-2*, *qHd3-1*, *Ghd7*, *Ghd8*, *OsPRR37*, *qHd2-1*, *qHd7b*, *EHd1*, and *Ghd7.1* have been identified to be the critical genetic factors on HD [11,12,13,14,15,16,17]. TGW, ETN, NSP, and SS are actually the yield component traits. TGW can also be viewed as the combined index of grain length, width, and thickness. QTLs, such as *qTGW2*, *qTGW3*, *qGW1*, *qKL3*, and *qKL7*, and genes, such as *GW2*, *GS3*, and *OsPPKL1*, have been identified to be the key determining factors on TGW [18,19,20,21,22]. A number of genes/QTLs, such as *qTN6-1*, *qTN7-1*, *MOC1*, *LAX*, *SPA*, *OsPILS6b*, and *qTN4*, have been proven to regulate ETN and improve the plant architecture [8,23,24,25,26]. NSP and SS together determine the number of grains per individual plant. As examples, *Gn1a*, *DEP1*, *qPSG1*, *GN4-1*, and *qPE9-1* have been found to be associated with NSP and grain number [27,28,29]. A number of QTLs on SS have been reported from a genome-wide association study [30] and near-isogenic lines derived from interspecific crosses [31].

Populations that can be used in genetic studies are many. Several criteria have been used to classify the genetic populations, among which are the number of parents, presence or absence of heterozygotes, homozygotes or heterozygotes of the parents, whether the selection was applied during population development, and the frequencies of alleles at each locus [32]. Bi-parental populations derived from two homozygous parents are mostly used in genetic studies, such as F_2_, recombination inbred lines (RILs), and doubled haploids [2,9,10,11,19]. Heterozygous genotypes have to be present in the populations in order to investigate the dominance-related effects and genetic basis of heterosis in hybrid rice breeding. However, individuals in conventional F_2_ and backcross populations cannot be maintained by self-propagation. To overcome the problem, the immortalized F_2_ and immortalized backcross populations have been proposed and used [33,34]. A QTL mapping method and the related software package applicable to immortalized populations have been reported. Multi-parental advanced inter-crossing populations have higher allelic and phenotypic diversity, and more recombination events, which are becoming more and more popular in plant genetic studies in recent years. A number of mapping populations derived from four and eight parental lines have been developed in rice and then used in linkage map construction and QTL mapping [35,36].

Chromosomal segment substitution lines (CSSLs) are useful for the precision mapping of QTLs and evaluation of inter-genic and intra-genic actions [37]. In the idealized situation, each CSS line in the population carries one single chromosomal segment from the donor parent, i.e., SSSL, which can be viewed as the isogenic line when compared with the background parent. It is well known that false positives frequently occur with bi-parental and multi-parental populations, and the mapping resolution is relatively low due to the high level of linkage disequilibrium [4]. Therefore, a large number of CSSLs/SSSLs have been developed in rice and used as independent genetic materials to validate the QTLs detected in conventional mapping populations [10,12,19,38,39,40]. SSSLs have the advantage of the efficient selection of candidate genes within a marker interval, and therefore, they can be further used in fine mapping and gene cloning for the validated QTLs. Reciprocal CSSLs/SSSLs allow the investigation of gene-by-background interactions. Hybridization of CSSLs/SSSLs with their background parents allows the investigation of the dominant effect as well at the donor segment of interest [32].

To our understanding, reports on the use of reciprocal SSSLs in QTL mapping and validation are still limited. In past years, we developed one bi-parental population consisting of 215 recombination inbred lines (RILs) from the inter-specific cross between *japonica* Asominori and *indica* IR24. In the meantime, we developed two introgression populations consisting of reciprocal SSSLs, which are denoted by AIS and IAS, respectively. In population AIS, each SSSL carries one donor segment from IR24 in the Asominori background; while in population IAS, each SSSL carries one donor segment from Asominori in the IR24 background. Our objectives in this study were (1) to conduct the preliminary QTL mapping on six yield-related traits in the RIL population, (2) to validate and identify the stable and novel QTLs by the AIS and IAS populations, and (3) to identify the SSSLs which are of value in the reciprocal improvement between *japonica* and *indica* varieties.

## 2. Results

### 2.1. Phenotypic Distribution, Correlation, and Analysis of Variance (ANOVA)

**Figure 1:**
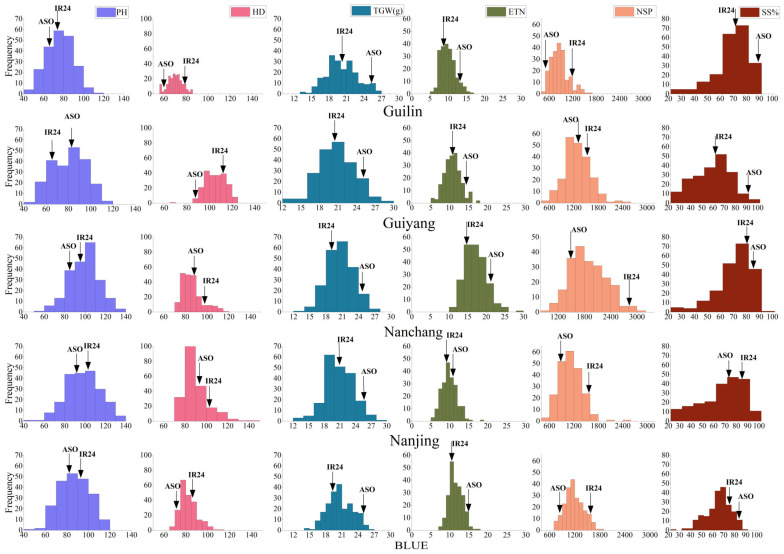
Frequency distributions of six yield-related traits in the rice (*Oryza sativa* L.) bi-parental RIL population. The two parents are represented by symbols IR24 and ASO at the top of each histogram. The six traits are denoted by PH: plant height; HD: heading date; TGW: thousand-grain weight; ETN: effective tiller number; NSP: number of spikelets per panicle; SS: seed set percentage. The four environments and BLUE values are denoted by GL: Guilin; GY: Guiyang: NC: Nanchang; NJ: Nanjing; and BL: BLUE.

Figure 1 shows the frequency distributions of six yield-related traits in the RIL population across the four environments. Best linear unbiased estimates (BLUEs) calculated from the four environments were included as well. Continuous distribution was observed for each trait, which is common for most quantitative traits. A clear difference occurred between the two parents. IR24 (*indica*) was taller than Asominori (*japonica*) in environments GL, NC, and NJ, but shorter in environment GY. The altitude of GY is about 1100 m above sea level, and the maximum temperature is between 25 and 28° C, which is significantly different from the other three locations. The temperate climate in GY makes it more suitable for growing *japonica* rice, which explains the taller PH and higher grain yield Supplementary Materials Data S1 of Asominori in comparison with IR24. IR24 exhibited higher phenotypic values on HD and NSP across the four environments and BLUEs. Asominori showed higher phenotypic values consistently on ETN and TWG. Asominori had higher SS in environments GL, GY, and NC, but lower SS in environment NJ. Transgressive segregations were observed in both directions for all traits in the RIL population.

Given in Table 1 are the correlation coefficients between the six traits in the three populations, calculated from the averaged phenotypic values across environments and replications. In the RIL population, PH showed positive correlations with SS and TGW and a negative correlation with ETN. In the AIS population, PH revealed positive correlations with HD and NSP and a negative correlation with ETN. In the IAS population, there was a positive correlation between PH and HD and a negative correlation between PH and ETN. In the RIL population, HD was negatively correlated with TGW and ETN. In reciprocal SSSL populations, there were no positive correlations between HD and other yield-related traits. However, NSP revealed a positive correlation with ETN but a negative correlation with TGW in the three populations. SS was positively correlated with TGW in the RIL and AIS populations, whereas no significant correlation was observed in the IAS population. Even though the three populations were derived from the same parents, they have much different genetic structures by allelic and genotypic frequencies, which explained the varied relationship in correlation observed in the three populations (Table 1).

Given on the left side of Table 2 are the estimates of variance components for the four sources of variations that were included in the ANOVA linear model; given on the right side of Table 2 are the two levels of heritability. The variance component quantifies the size of each source of variation; heritability quantifies the proportion of genetic variance in phenotypic variance. In the RIL population, ANOVA on the multi-environmental phenotypic data revealed highly significant variations from genotypes, environments, and genotype-by-environment (GE) interactions for the six traits (Table 2). However, genetic variance in the population was much higher than the variances from the other two sources of variation. Variance components estimated from ANOVA were then used to calculate the two levels of heritability, which are given on the right side of Table 2. The heritability of the six traits was between 0.31 and 0.77 at the plot level and between 0.67 and 0.94 at the phenotypic mean level. PH had the highest and SS had the lowest heritability among the six traits. In populations AIS and IAS, results from ANOVA indicated there were significant genetic variations from PH, HD, TGW, and SS and significant environmental variation from all traits. Even though some GE interactions were significant, variances from the interactions were much smaller than those from genotypes and environments. When the three populations were considered together, the heritability of the six traits was between 0.05 and 0.79 at the plot level and between 0.25 and 0.96 at the phenotypic mean level. TGW had the highest heritability in population AIS, followed by PH and HD. PH had the highest heritability in population IAS, followed by HD and TGW (Table 2). The heritability of these traits was located in the medium to high regions by quantitative genetics, indicating that they were suitable for QTL mapping and further genetic investigations.

### 2.2. QTLs Identified for Six Yield-Related Traits in the RIL Population

By using the BIP functionality in the QTL IciMapping V4.2, a total of 18 stable QTLs were identified for the six traits, 4 for PH, 6 for HD, 5 for TGW, and 1 each for ETN, NSP, and SS (Table 3). The information for all QTLs can be found in Table S1. The 18 stable QTLs were detected in two or more environments and distributed on 8 of the 12 chromosomes in rice. Most of the stable QTLs were identified by BLUE values as well. More QTLs were identified for PH, HD, and TGW due to their higher heritability, and fewer QTLs were identified for ETN, NSP, and SS due to their lower heritability (Table 2).

QTLs identified for PH, i.e., *qPH1, qPH2, qPH3-1*, and *qPH3-2*, had LOD scores ranging from 2.51 to 8.52 and explained 2.66~17.88% of phenotypic variance (PVE) (Table 3). IR24 was consistently taller than Asominori (Figure 1). The additive effects from QTL mapping indicated the alleles from IR24 increased PH at *qPH2, qPH3-1*, and *qPH3-2*, but the allele from IR24 decreased PH at *qPH1* (Table 3). QTLs identified for HD, i.e., *qHD3-1*, *qHD3-2, qHD5, qHD6, qHD8,* and *qHD12*, had LOD scores ranging from 3.02 to 15.35, and PVE ranged from 2.92 to 20.00%. IR24 was later on HD in comparison with Asominori (Figure 1). The alleles from IR24 delayed HD at *qHD3-1*, *qHD3-2, qHD5, qHD8,* and *qHD12*, but the allele from IR24 reduced HD at *qHD6* (Table 3). QTLs identified for TGW, i.e., *qTGW2-1*, *qTGW2-2*, *qTGW3-1*, *qTGW3-2*, and *qTGW5*, had LOD scores ranging from 2.50 to 7.20, and PVE ranged from 4.83 to 10.72%. Asominori consistently had higher TGW than IR24 (Figure 1). The alleles from Asominori increased TGW at *qTGW2-1*, *qTGW2-2*, *qTGW3-2*, and *qTGW5,* but the allele from Asominori decreased TGW at *qTGW3-1* (Table 3). The dispersed distribution of positive alleles between the two parents explained the transgressive segregation observed in phenotypic distributions of PH, HD, and TGW (Figure 1). Transgressive segregations were also observed for ETN, NSP, and SS. Due to their low heritability (Table 2), there was only one QTL identified for each of the three traits, and the LOD and PVE were lower in comparison with QTLs on PH, HD, and TGW (Table 3). It was expected that more QTLs would be detected if a lower LOD threshold was used.

### 2.3. Evaluation of QTL-by-Environment Interactions in the RIL Population

The 18 stable QTLs as shown in Table 3 were repeatedly identified by the MET functionality in the QTL IciMapping V4.2. In functionality MET, combined QTL mapping was conducted across the four environments; therefore, each detected QTL had one estimated chromosomal position and one estimated genetic effect (Table 4). Most of the estimated positions and additive effects from the combined mapping were located in the respective ranges as given in Table 3. However, two LOD scores and two PVEs are given by the combined mapping method. As shown in Table 4, LOD was used to test the significance of overall genetic variation, LOD (AbyE) was used to test the significance of QTL-by-environment interactions, PVE was used to quantify the overall genetic variation, and PVE (AbyE) was used to quantify the variation of QTL-by-environment interactions. For most QTLs, for example, *qPH3-1*, *qHD5*, and *qTGW2-1* (Table 4), LOD (AbyE) was much lower than the overall LOD score, and PVE (AbyE) was much lower than the overall PVE, indicating their interactions with environments were less significant. These results were consistent with the lower variance components of GE as given in Table 2. The information for all QTLs detected by the MET functionality can be found in Table S2.

### 2.4. Validation of the Stable QTLs on Yield-Related Traits by Reciprocal SSSL Populations

Using the CSL functionality in QTL IciMapping V4.2, 8 of the 18 stable QTLs (Table 3, Table 4) were validated in populations AIS and/or IAS, 3 for HD and 1 each for the other five yield-related traits (Table 5, Figure S1). *qHD3-1* and *qSS1* were validated by both directions of SSSLs. *qPH3-1* was validated in the AIS population at genetic position 51.33 cM with an LOD score of 2.69~3.24, PVE of 14.54~17.01%, and additive effect of 4.89~5.35. *qHD3-1, qHD6*, and *qHD8* were validated by the AIS population, at genetic positions 1.00, 42.23, and 37.45 cM, respectively. The LOD score of the three QTLs was in the ranges of 6.07~13.53, 3.47~16.20, and 8.21~11.38; PVE was in the ranges of 9.66~30.10%, 7.34~38.45%, and 17.68~22.23%; and additive effect was estimated to be in the ranges of 2.91~4.87, 5.10~1.35, and −6.26~−3.58, respectively. The information for all QTLs detected by the CSL functionality can be found in Table S3.

**Figure 2:**
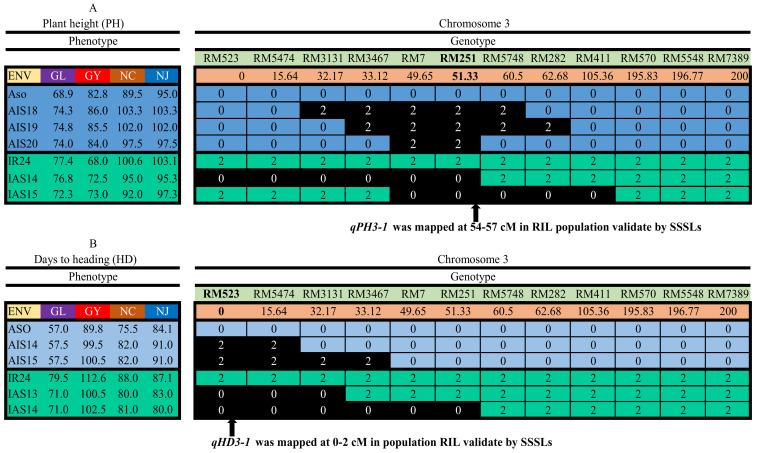
Validation of two QTLs on chromosome 3, one for PH and one for HD, i.e., *qPH3-1* (**A**) and *qHD3-1* (**B**), by reciprocal SSSLs. Indicated on the left side are phenotypic values; on the right side are marker genotypes. Chromosomal segments of Asominori are denoted by 0, and IR24 denoted by 2.

Out of the 18 stable QTLs identified in the RIL population, *qPH3-1*, *qHD3-1*, *qHD6*, *qHD8*, *qTGW2-2*, and *qNSP6* were further validated in population AIS, and *qETN11* and *qSS1* were further validated in population IAS on the same marker positions (Table 5, Figure 2). As two examples, *qPH3-1* was located in marker interval RM251-RM5748 on chr. 3, and its physical positions were 9949856:12327987 bps; *qHD3-1* was located in marker interval RM523-RM5474 on chr. 3, and its physical positions were 1320598:3804270 bps (Table 3, Figure S2).

### 2.5. Validation of Two Linked QTLs for PH and HD by Reciprocal SSSLs

Two QTLs, i.e., *qPH3-1* for PH and *qHD3-1* for HD, were used to further demonstrate the QTL validation by reciprocal SSSLs. *qPH3-1* and *qHD3-1* were located on the same chromosome, with a genetic distance of around 54 cM based on the combined mapping results of the MET functionality (Table 4). In Figure 2, the phenotypic values of some selected SSSLs and the background parent are indicated on the left side, and their marker genotypes are indicated on the right side. Chromosomal segments of Asominori are denoted by 0, and those of IR24 are denoted by 2. The genetic positions of markers on chromosome 3 are also indicated in Figure 2.

It can be observed from the left side of Figure 2A that three SSSLs, i.e., AIS18, AIS19, and AIS20, were consistently taller across four environments than the background parent Asominori. Obviously, the allele from Asominori reduced PH. *qPH3-1* was located on the donor segments carried in the three SSSLs (Table 4, Figure 2A). AIS20 carries the shortest donor segment (i.e., marker interval from RM7 to RM251) from Asominori, which reduced PH. There are two SSSLs from the other direction, i.e., IAS14 and IAS15, which had shorter PH than the background parent IR24 across three environments. IR24 was consistently taller than Asominori in the three environments. The allele from IR24 increased PH in environments GL, NC, and NJ. The donor segments from five SSSLs overlapped on the marker interval from RM7 to RM251 (Figure 2A), where *qPH3-1* was located. The results above indicated that *qPH3-1* was validated by both directions of SSSLs in three environments. It may have the opposite genetic effect on PH in the other environment.

From the left side of Figure 2B, it can be observed that two SSSLs, i.e., AIS14 and AIS15, were consistently later across four environments than the background parent Asominori. Obviously, the allele from Asominori reduced HD. *qHD3-1* was located on the donor segments carried in the two SSSLs (Table 4, Figure 2). AIS14 carries the shorter donor segment (i.e., marker interval from RM523 to RM5474) from IR24, which delayed HD. There are two SSSLs from the other direction, i.e., IAS13 and IAS14, which were consistently earlier across four environments than the background parent IR24. The allele from IR24 reduced HD. The donor segments from the four SSSLs overlapped on the marker interval from RM523 to RM5474 (Figure 2B), where *qHD3-1* was located. The results above indicated that *qHD3-1* was validated by both directions of SSSLs. The validation by reciprocal SSSLs for other QTLs can be found in Table S4.

## 3. Discussion

In conventional QTL mapping studies, various bi-parental populations have been used. However, mapping resolution is relatively low, and the false discovery rate is high with conventional mapping populations [4,32]. In addition, environmental conditions can significantly affect the accuracy of QTL mapping, making it challenging to uncover the genetic architecture of quantitative traits. For further genetic studies and breeding applications, QTLs detected in those populations have to be validated by independent genetic materials. CSSLs are such materials, being useful for QTL validation and the evaluation of inter-genic and intra-genic genetic actions [12,37]. Each CSSL can be treated as the isogenic line with the background parent when it carries a single segment from the donor parent, i.e., SSSL. Reciprocal CSSLs/SSSLs allow the investigation of QTL-by-background interactions. The hybrids of these lines with their background parents allow the investigation of the dominant effects as well [32]. In this study, we present an approach by using bi-parental RILs for QTL identification and reciprocal SSSLs for QTL validation on yield-related traits in rice. From previous QTL mapping studies, it appears that SSSLs have not been as widely used as expected. To our understanding, the long and tedious procedure that is required for population development could be the major reason. For instance, it took us more than 5 years to convert some existing CSSLs into SSSLs that were used in this study. The use of reciprocal SSSLs in QTL validation can be treated as the innovative aspect of this study.

In the RIL population, 18 stable QTLs were detected for six yield-related traits, 8 of which were validated by reciprocal SSSLs. Some QTLs were located on the same chromosomes. For example, *qSS1* and *qPH1* were located on chromosome 1; *qPH3-1* and *qHD3-1* were located on chromosome 3. These results suggested that the correlation between PH, HD, and SS observed in RILs (Table 1) was due to genetic linkage rather than pleiotropic effects. PH is a major determinant factor for plant architecture and biomass and is therefore highly associated with grain yield. In the present study, *qPH3-1* was identified to be a stable QTL in population RIL, which was also validated by reciprocal SSSLs. It was located in marker interval RM251-RM5748 at the genetic position of 54~57 cM on the linkage map and the physical position of 9949856:12327987 bps on the physical map (Table 3). Previous studies also reported QTLs on PH located at 32.11~37.81, 3.48.3~50.7 MBs on chromosome 3 [9,10] (Table 5). But their physical positions were not close to *qPH3-1*, indicating *qPH3-1* could be novel to this study. In another study, one QTL on PH was reported on chromosome 3 with pleiotropic effects on other yield-related traits [42]. IR24 is an *indica* cultivar that is tall in PH and mostly cultivated in tropical and sub-tropical regions. IAS14 and IAS15 have alleles from Asominori, which can reduce PH and therefore prevent IR24 from lodging. On the other hand, AIS18, AIS19, and AIS20 have alleles from IR24 that can be used to increase PH and therefore can possibly increase biomass and grain yield in Asominori (Figure 2A). HD determines rice adaptation, cropping seasons, and maturity, and contributes to grain yield. In this study, we identified six stable QTLs on HD in bi-parental RILs and validated three of them in reciprocal SSSL populations, i.e., *qHD3-1*, *qHD6*, and *qHD8*, in marker intervals RM411-RM7097, RM6863-RM72, and RM557-RM13 at genetic positions 3.18~16.44, 3.73~6.28, and 7.06~15.35 cM, respectively. The positions of these QTLs overlapped with some QTLs reported from previous studies [12,15,41] (Table 5). *HD6* was reported for photoperiod sensitivity on the long arm of chromosome 3 [41]. Asominori is suitable for temperate regions (e.g., northern China). Two SSSLs, i.e., AIS14 and AIS15, can prolong the HD of Asominori; on the other hand, the other two SSSLs, i.e., IAS13 and IAS14, can be used to reduce the HD of IR24 (Figure 2B).

TGW is an integrated index of grain length, width, and thickness. *qTGW2-2* was located in the marker interval RM6~RM425 at the genetic position of 143.95 cM, which was validated by reciprocal SSSLs. One previous study also reported a QTL on TGW on the long arm of chromosome 2 near marker RM6 [18] (Table 5). On the same chromosome, one QTL i.e., *GW2*, has been cloned and characterized. The loss of the *GW2* function accelerated the rate of grain filling, resulting in enhanced grain width, weight, and yield [20]. ETN, NSP, and SS are the other yield components. A stable and validated QTL for ETN, i.e., *qETN11*, was located at a genetic position of 19.35 cM between the flanking markers RM4 and RM6288. Previous studies reported QTLs on chromosome 11 but far from the marker interval [8]. We suspect there are some genes near *qETN11* with functions similar to those of *SPA, LAX*, and *Monoculm1*, which have been proven to affect the number of tillers and regulate branching in rice [23,24]. On the linkage map, *qNSP6* was located at the genetic location of 59.92 cM within the marker interval RM6818~RM541. *qSS1* was located within marker interval RM14~RM5410 at genetic position 137~150 cM, which was further validated by reciprocal SSSLs. Earlier researchers identified QTLs on the same chromosomes for NSP and SS, respectively [27,29,30] (Table 5).

The QTL information provided in this study can be useful in enhancing the six yield-related traits in rice breeding. DNA markers associated with these QTLs can be used to screen and select individual plants with desired genotypes in early generations, saving time and resources. Reciprocal SSSLs provide significant benefits in rice breeding by validation of QTLs, allowing for the precise selection of donor genes leading to the desirable phenotypes [12,32,37]. Genetic materials used in this study help to transfer beneficial alleles between *japonica* and *indica* [38]. The mutual exchange of beneficial alleles between *japonica* and *indica* has the potential to develop new rice varieties with superior attributes. The SSSLs used in this study can be further utilized for the fine mapping and cloning of the candidate genes of the detected QTLs.

The three populations used in this study were genotyped by 143 SSR markers showing polymorphism between two parental lines Asominori and IR24. We understand that genotyping by SSR markers has become less popular in recent years of genetic studies. Instead, genotyping by next-generation sequencing and high-density SNP chips are more frequently used. In comparison with genetic mapping with natural populations, linkage disequilibrium (LD) is high, and the number of crossing over is rather limited in bi-parental populations. For this reason, linkage maps at the medium level of density can meet the requirements of QTL linkage mapping, and the genomic coverage is more important. The 143 SSR markers used in this study cover the whole rice genome of 12 chromosomes. We trust that the chance of missing large-effect QTLs would be low. In fact, the three populations have been genotyped by a re-sequencing technology called 2b-RAD [43]. A linkage map with several thousands of SNP markers has been constructed. We are combining the SNP markers with SSR markers to make the combined map, after which more QTL mapping, fine mapping, and validation studies will be conducted and reported.

## 4. Materials and Methods

### 4.1. Genetic Populations, Genotyping, and Linkage Map

**Figure 3:**
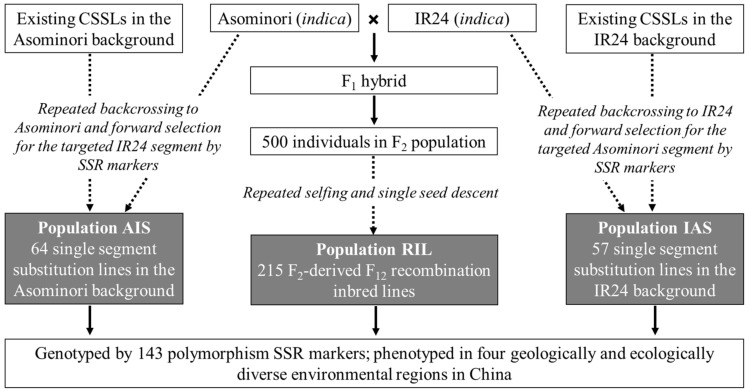
The development diagram of the RIL and reciprocal SSSL populations from the same two parents.

In this study, 215 F_12_ RILs in rice (*Oryza sativa* L.) were used for the preliminary investigation of QTLs on six yield-related traits (Figure 3). Asominori from *japonica* and IR24 from *indica* were the two parental materials. The F_12_ advanced lines were developed through the single seed descent (SSD) method from generations F_2_ to F_12_ [44]. The use of SSD can maximize the effective population size and avoid selection during population development. Due to the high diversity between the two parents, segregation was still observed in the filial generation of F10. Therefore, we performed two additional rounds of selfing to ensure the acquisition of pure and uniform recombination inbred lines, which were called F_2_-derived F_12_ RILs. The reciprocal SSSLs were made by two directions of one to three times of backcrosses between existing CSSLs and the respective background parents (Figure 3). Proportions of donor genomes in existing CSSLs are varied, which was the reason for performing the different numbers of backcrosses. Donor segments were chosen based on marker information observed in backcrossing generations. In the end, 64 SSSLs were obtained in the background of Asominori (called AIS), and 57 SSSLs were obtained in the background of IR24 (called IAS) (Figure 3) [45]. Genotyping of these RILs and SSSLs was performed using SSR markers. A linkage map was constructed from the RIL population, with a total length of 1,472.31 cM. The 143 polymorphism SSR markers were distributed rather evenly on the 12 chromosomes.

### 4.2. Field Experiments and Trait Measurement

The three populations, i.e., one RIL and two directions of SSSLs, and their parental lines were grown in four diverse locations in China, i.e., Guilin (GL), Guiyang (GY), Nanchang (NC), and Nanjing (NJ). The materials were grown from May to November 2013. A randomized complete block design was applied in field experiments, with two replicates in each location. Each plot consisted of four rows, each with ten plants. Throughout the growing season, the field was managed by conventional agricultural practices. Six yield-related traits were measured in the experiment, i.e., PH, HD, TGW, ETN, NSP, and SS. At the grain-filling stage, five representative plants were chosen in the middle rows of each plot. The heights of the five main panicles were measured, and their average value was used as the phenotypic value of PH. HD was recorded when the main panicle was observed for 90% of individual plants in each plot. HD was counted from the transplanting date. Phenotypical traits related to grains were evaluated by the SC-G rice grain image analysis system developed by Hangzhou WSeen Detection Technology Co., Ltd., Hangzhou, China. This system is able to distinguish individual grains when they are randomly spread on a flat-bed surface. The grown plants were harvested individually at the maturity stage. Panicles from individual plants bearing 5 or more filled grains were counted as ETN. NSP was counted by individual panicle. Total ears and seeding ears were counted manually, and then SS was calculated.

### 4.3. Phenotypic Data Analysis and the Estimation of Heritability

Phenotypic data analysis was completed by the AOV functionality in QTL IciMapping V4.2 [46]. Pearson’s correlation coefficient was calculated and used to quantify the linear relationship between the six traits. ANOVA was conducted to test the significance of various sources of variation, estimate variance components, and calculate the best linear unbiased estimates of the tested lines and heritability values of the yield-related traits [32]. The observed phenotype comprised the overall mean and various effects originated from replications (or blocks) per environment, genotypes, environments, genotype-by-environment interactions (GEs), and random errors. The following linear equation was used in ANOVA, where *y_ijk_* represents the measured value of a specific trait of interest for the *i*th line in the *j*th environment and the *k*th replication; *i* = 1, 2,…, *n* (in this study, *n* = 215 for the RIL population, 64 for the AIS population, and 57 for the IAS population); *j* = 1, 2,…, *e* (*e* = 4 in this study); *k* = 1, 2,…, *r* (*r* = 2 in this study); *µ* is the overall mean; *R_k/j_* is the effect of the *k*th replication in the *j*th environment; *G_i_* is the genotypic effect of the *i*th line; *E_j_* is effect of the *j*th environment; *GE_ij_* is effect of the interaction between the *i*th line and the *j*th environment; and $\varepsilon_{ijk}$ is the effect of random error, which is assumed to follow a normal distribution with a mean of zero and variance of $\sigma_{\varepsilon }^{2}$.

$$y_{ijk}=µ+R_{k/j}+G_{i}+E_{j}+{GE}_{ij}+\varepsilon_{ijk},\;\varepsilon_{ijk}\sim N(0,\sigma_{\varepsilon }^{2})$$

Based on the above linear model, ANOVA was conducted to partition the total degree of freedom and the total sum of squares into the respective components. Mean squares obtained from ANOVA were then used to calculate the unbiased estimates of variance components included in the linear model. Given below are the equations for the estimation of genetic variance ($\sigma_{G}^{2}$), GE interaction variance ($\sigma_{GE}^{2}$), and error variance ($\sigma_{\varepsilon }^{2}$).

$${\hat{\sigma }}_{G}^{2}=\frac{1}{e\times r}{(MS}_{G}-{MS}_{\varepsilon }),{\hat{\sigma }}_{GE}^{2}=\frac{1}{r}{(MS}_{GE}-{MS}_{\varepsilon }),{\hat{\sigma }}_{\varepsilon }^{2}={MS}_{\varepsilon }$$

ANOVA was also conducted for each environment, and the error variances for the four environments were calculated separately, based on which the best linear unbiased estimate (BLUE) of each genotype was acquired [32]. BLUE is the weighted average across environments, which gives the minimum sampling variance in all unbiased estimates of genotypic value. The equation for calculating BLUE is given below.

$${\hat{\mu }}_{i}=\sum_{j=1,\ldots ,\;e}w_{j\;}{\bar{y}}_{ij+},\;\mathrm{where}\;{\bar{y}}_{ij+}=\frac{1}{r}\sum_{k=1,\ldots ,\;r}y_{ijk},\;\;\;\;\;\;\;w_{j}=\frac{\frac{1}{\sigma_{\varepsilon j}^{2}}}{\frac{1}{\sigma_{\varepsilon 1}^{2}}+\frac{1}{\sigma_{\varepsilon 2}^{2}}+\ldots +\frac{1}{\sigma_{\varepsilon e}^{2}}},\;\mathrm{and}\;\sigma_{\varepsilon j}^{2}\;\mathrm{is}\;\mathrm{the}\;\mathrm{random}\;\mathrm{error}\;\mathrm{variance}\;\mathrm{in}\;\mathrm{the}\;j\mathrm{th}\;\mathrm{environment}.$$

Heritability in the broad sense is equal to the proportion of genetic to phenotypic variance in the population. There is a consensus that when calculating heritability, it is not appropriate to consider environmental variance. In multi-environmental trials, the phenotypic variance per plot is the summation of genetic variance, genotype-by-environment interaction variance, and error variance, i.e.,${\;\sigma }_{P}^{2}=\sigma_{G}^{2}+\sigma_{GE}^{2}+\sigma_{\varepsilon }^{2}$. However, genetic analysis and breeding selection are always based on means across replications for single-environment trials and means across replications and environments for multi-environment trials. As far as the multi-environment trials are concerned, the phenotypic variance based on the means across replications and environments is ${\;\sigma }_{\bar{P}}^{2}=\sigma_{G}^{2}+\frac{1}{e}\sigma_{GE}^{2}+\frac{1}{er}\sigma_{\varepsilon }^{2}$. The following two equations are used to calculate the heritability at the plot level and the heritability at the mean level, respectively.

$$H_{P}^{2}=\frac{\sigma_{G}^{2}}{\sigma_{P}^{2}}=\frac{\sigma_{G}^{2}}{\sigma_{G}^{2}+\sigma_{GE}^{2}+\sigma_{\varepsilon }^{2}}\;\mathrm{and}\;H_{\bar{P}}^{2}=\frac{\sigma_{G}^{2}}{\sigma_{\bar{P}}^{2}}=\frac{\sigma_{G}^{2}}{\sigma_{G}^{2}+\frac{1}{e}\sigma_{GE}^{2}+\frac{1}{er}\sigma_{\varepsilon }^{2}}$$

### 4.4. QTL Mapping Methods in the RIL and Reciprocal SSSL Populations

Three functionalities in the QTL IciMapping package V4.2 were used in this study for QTL mapping [46]. Functionalities BIP and MET were applied in the RIL population, and CSL was applied in the AIS and IAS populations. In QTL mapping, BLUE values were considered as one separate environment. BIP was used for QTL mapping in each environment, and MET was used for combined QTL mapping across the four environments. The inclusive composite interval mapping (ICIM) method was employed in functionalities BIP [42] and MET [47]. It has been proved that ICIM has high detection power through background genetic control and can be easily extended to mapping QTLs with epistasis and environmental interactions. The scanning step in ICIM was set at 0.2 cM. In stepwise regression, the probabilities for variable inclusion and exclusion were set at 0.001 and 0.002, respectively. For all QTL mapping analyses, i.e., BIP, MET, and CSL, the threshold LOD score was set at 2.5. QTL names were assigned to indicate the information on the located chromosomes, the number of QTLs, and the name of a particular trait. One QTL was considered to be stable in a population if detected in two or more environments within a range of 15 cM on the same chromosome. When one QTL was identified by BIP and MET analysis at the same marker position and was also identified by CSL analysis, the QTL was considered to be validated.

### 4.5. Physical Positions of SSR Markers and Genomic Visualization of Markers and QTLs

Both forward and reverse primer sequences were extracted from the Gramene database [48] for the 143 polymorphism SSR markers which were used in genotyping RILs and SSSLs and linkage map construction. Using these sequences, BLAST was conducted in the Rice Annotation Project (RAP-DB) (https://rapdb.dna.affrc.go.jp/tools/blast, accessed on 20 June 2024), and therefore, the physical locations of these SSR markers were acquired. The shinyCircos-V2.0 was used to visualize the physical and linkage positions of markers and QTLs on the rice genome [49].

## Figures and Tables

**Table 1:** Correlation coefficients across the four environments between the six yield-related traits in the RIL and reciprocal SSSL populations.

Population	Trait	Trait				
		PH	HD	NSP	SS	TGW
RIL	HD	0.0269 ns				
	NSP	0.1253 ns	−0.0786 ns			
	SS	0.2108 **	−0.0278 ns	0.0381 ns		
	TGW	0.2404 ***	−0.1788 **	−0.2968 ***	0.2519 ***	
	ETN	−0.2524 ***	−0.2821 ***	0.1355 *	0.0088 ns	−0.1234 ns
AIS	HD	0.5006 ***				
	NSP	0.3245 **	0.2382 ns			
	SS	0.1209 ns	0.0846 ns	−0.0422 ns		
	TGW	0.0080 ns	−0.1884 ns	−0.3176 **	0.5028 ***	
	ETN	−0.3906 **	−0.2123 ns	0.4161 ***	−0.1006 ns	−0.2667 *
IAS	HD	0.3301 *				
	NSP	−0.1597 ns	−0.1537 ns			
	SS	0.1907 ns	−0.1937 ns	−0.1716 ns		
	TGW	−0.1635 ns	−0.1528 ns	−0.5439 ***	0.0444 ns	
	ETN	−0.4406 ***	−0.1418 ns	0.4891 ***	0.2823 *	−0.1543 ns

*, **, and *** represent the significant levels at 0.05, 0.01, and 0.001, respectively; ns stands for non-significant. The six traits are denoted by PH: plant height; HD: heading date; TGW: thousand-grain weight; ETN: effective tiller number; NSP: number of spikelets per panicle; SS: seed set percentage.

**Table 2:** Variance components and heritability in the broad sense of the six yield-related traits in the RIL and reciprocal SSSL populations.

Population	Trait	Variance Components				Heritability	
		Genotype	Environment	GE Interaction	Random Error	Plot Level	Genotypic Mean Level
RIL	PH	189.17 ***	140.66 ***	35.57 ***	21.21	0.77	0.94
	HD	62.45 ***	206.86 ***	26.02 ***	6.07	0.66	0.90
	TGW	5.39 ***	0.09 ***	1.78 ***	0.95	0.66	0.90
	ETN	2.60 ***	12.22 ***	1.01 ***	4.04	0.34	0.78
	NSP	48,804.04 ***	192,351.84 ***	23,251.20 ***	60,185.55	0.37	0.78
	SS	114.45 ***	41.54 ***	6.05 ***	197.71	0.31	0.67
AIS	PH	28.28 ***	97.99 ***	7.78 ns	16.16	0.54	0.88
	HD	4.03 ***	196.51 ***	4.08 ***	1.77	0.41	0.76
	TGW	1.30 ***	0.24 ***	0.22 ns	0.54	0.63	0.91
	ETN	0.60 ns	16.54 ***	0.00 ns	10.01	0.06	0.32
	NSP	2617.29 ns	126,986.19 ***	6615.26 ns	48,633.88	0.05	0.25
	SS	7.63 ***	19.05 ***	19.37 ***	26.19	0.14	0.48
IAS	PH	59.96 ***	265.84 ***	4.06 ns	11.75	0.79	0.96
	HD	6.28 ***	213.46 ***	1.79 ***	1.57	0.65	0.91
	TGW	1.35 ***	0.52 ***	0.42 ***	0.65	0.56	0.88
	ETN	0.60 ns	14.31 ***	0.42 ns	2.76	0.16	0.57
	NSP	10,910.70 ns	378,504.78 ***	378,504.78 ns	79,878.49	0.11	0.46
	SS	11.78 ***	130.52 ***	16.94 ***	17.17	0.26	0.65

*** represent the significant levels 0.001; ns stands for non-significant. PH: plant height; HD: heading date; TGW: thousand-grain weight; ETN: effective tiller number; NSP: number of spikelets per panicle; SS: seed set percentage.

**Table 3:** QTLs identified for the six yield-related traits in the rice (*Oryza sativa* L.) RIL population.

QTL Name ^a^	Chr.	Environment	Genetic Position (cM) ^b^	Left Marker ^c1^	Right Marker ^c2^	Physical Position (bp) ^d^	^e^ LOD	^f^ PVE (%)	^g^ ADD
*qPH1*	1	GY, NC	97~97	RM1003	RM486	33477070~34956587	2.51~3.77	2.66~4.3	−4.13~−3.15
*qPH2*	2	NJ, GL, GY, BL	129~134	RM6	RM425	29585867~32303944	3.10~8.52	10.66~17.4	8.37~9.76
*qPH3-1*	3	NC, NJ, BL	54~57	RM251	RM5748	9949856~12327987	3.53~4.57	3.73~7.21	4.46~6.15
*qPH3-2*	3	NC, NJ, BL	110~114	RM411	RM7097	21430854~26881168	2.67~5.02	4.06~10.87	4.71~4.78
*qHD3-1*	3	GL, GY, NC, NJ, BL	0~2	RM523	RM5474	1320598~3804270	3.18~16.4	3.55~15.47	1.68~3.9
*qHD3-2*	3	GL, GY, NC, NJ, BL	163~166	RM8269	RM448	31339287~31399595	4.79~8.80	4.68~11.56	2.22~3.10
*qHD5*	5	GL, GY	1~16	RM13	RM413	2011198~2212829	3.02~3.72	2.92~3.99	1.31~1.70
*qHD6*	6	NC, NJ, GY, GL, BL	48~55	RM557	RM136	8552551~8752551	3.73~6.28	6.82~11.61	−1.98~−4.93
*qHD8*	8	NC, NJ, GY, GL, BL	31~37	RM6863	RM72	2012432~2212432	7.06~15.3	9.21~20.00	6.70~−1.97
*qHD12*	12	GL, GY, NC, BL	27~32	RM6296	RM7102	3201718~12952364	2.63~5.33	5.18~7.08	1.54~2.78
*qTGW2-1*	2	GL, BL	22~26	RM211	RM71	8560434~8760434	2.50~3.08	7.63~8.22	−0.97~−0.95
*qTGW2-2*	2	GL, GY	133~136	RM6	RM425	29585849~32303935	4.17~7.20	5.20~7.20	−0.87~−0.75
*qTGW3-1*	3	NC, GY, BL	112~117	RM411	RM7097	21430825~26881139	3.98~6.02	4.83~9.86	0.77~0.97
*qTGW3-2*	3	GL, NC, BL	183~187	RM448	RM570	31399595~35595760	4.35~5.44	8.97~10.72	−1.09~−0.92
*qTGW5*	5	GL, GY, NC, BL	15~16	RM405	RM430	3073440~18753934	5.91~6.53	5.47~6.63	-096~−0.79
*qETN11*	11	NJ, BL	16~19	RM4	RM6288	932168~2166293	3.36~3.53	3.10~3.62	−0.57~−0.46
*qNSP6*	6	NC, NJ, GL, BL	61~79	RM6818	RM541	16582523~19514548	3.73~6.28	6.82~11.61	65.62~144.85
*qSS1*	1	GY, BL	137~150	RM14	RM5410	35477070~41094758	3.17~4.10	9.03~7.22	4.11~−8.26

^a^ QTLs identified by peaks on the LOD profile which are over the threshold value at least in one environmental condition; ^b^ genetic position in centimorgans (cM); names of left ^c1^ and right ^c2^ markers; ^d^ physical positions of left and right markers; ^e^ LOD profile peak values which exceed the threshold value; ^f^ the phenotypic variance explained (PVE) by QTL; ^g^ a positive value indicates that the allele from IR24 leads to an increase in phenotype, while a negative value indicates that the allele from Asominori leads to an increase in phenotypic values. The four environments and BLUE values are denoted by GL: Guilin; GY: Guiyang: NC: Nanchang; NJ: Nanjing; and BL: BLUE.

**Table 4:** QTLs identified by the combined mapping method across the four environments in the RIL population.

QTL	Position	Left Marker	Right Marker	^a1^ LOD	^a2^ LOD(AbyE)	^b1^ PVE	^b2^ PVE(AbyE)	^c^ Add	^d1^ LeftCI	^d2^ RightCI
*qPH1*	97	RM1003	RM486	10.56	0.55	3.07	0.18	−2.84	96.5	97.5
*qPH2*	138	RM6	RM425	3.26	3.10	0.73	0.66	0.44	132.5	151.5
*qPH3-1*	55	RM251	RM5748	17.33	0.21	5.70	0.17	4.34	52.5	58.5
*qPH3-2*	111	RM411	RM7097	18.42	0.99	5.81	0.06	3.99	105.5	115.5
*qHD3-1*	1	RM523	RM5474	36.84	6.47	7.99	0.50	2.60	0	2.5
*qHD3-2*	164	RM8269	RM448	36.64	4.53	7.92	0.08	2.64	163.5	164.5
*qHD5*	1	RM13	RM413	5.16	1.79	0.87	0.1	0.83	0	4.5
*qHD6*	49	RM557	RM136	31.36	13.79	7.76	3.96	−1.95	47.5	50.5
*qHD8*	35	RM6863	RM72	56.01	2.47	15.38	1.43	−3.51	32.5	36.5
*qHD12*	29	RM6296	RM7102	15.70	1.09	3.66	0.13	1.76	21.5	35.5
*qTGW2-1*	34	RM211	RM71	7.28	0.72	2.61	0.06	−0.41	21.5	43.5
*qTGW2-2*	133	RM6	RM425	9.87	2.74	3.43	0.87	−0.41	126.5	137.5
*qTGW3-1*	113	RM411	RM7097	15.42	1.32	5.76	0.26	0.60	107.5	117.5
*qTGW3-2*	187	RM448	RM570	15.05	1.39	5.60	0.07	−0.61	180.5	192.5
*qTGW5*	16	RM405	RM430	26.18	2.37	9.17	0.32	−0.77	14.5	18.5
*qETN11*	1	RM286	RM4	7.38	1.56	2.47	0.45	−0.33	0	5.5
*qNSP6*	61	RM6818	RM541	17.23	3.69	7.78	2.12	67.1	60.5	65.5
*qSS1*	150	RM14	RM5410	3.31	2.12	2.73	1.93	−1.14	139.5	151

^a1^ Test statistic for the significance of overall genetic variation; ^a2^ test statistic for the significance of QTL-by-environment interaction; ^b1^ percentage of phenotypic variance explained by overall genetic variation; ^b2^ percentage of phenotypic variance explained by QTL-by-environment interaction; ^c^ estimated additive effect; ^d1^ left side of the one-LOD drop confidence interval; ^d2^ right side of the one-LOD drop confidence interval.

**Table 5:** The yield-related QTLs validated by reciprocal SSSL populations.

QTL	Environment	Marker Name	Chr.	Pos. (cM)	LOD ^a^	PVE (%) ^b^	ADD ^c^	SSSL Population	Previously Published QTLs/Genes
*qPH3-1*	NC, NJ	RM251	3	51.33	2.69~3.24	14.54~17.01	4.89~5.35	AIS	*qPH3.1, qPH3b* [9,10]
*qHD3-1*	GY, NC, NJ, BL	RM523	3	0	6.07~13.50	9.66~30.10	2.91~4.87	AIS, IAS	*qHd3-1* [12]
*qHD6*	NC, NJ, BL	RM557	6	42.23	3.47~16.20	7.34~38.45	5.10~1.35	AIS	*qHD6a, qHD6b, HD6* [15,41]
*qHD8*	NC, NJ, BL	RM72	8	37.45	8.21~11.38	17.68~22.23	−6.26~3.58	AIS	*qHD8a* [15]
*qTGW2-2*	GY, NC	RM425	2	143.95	2.99~2.64	19.40~15.30	1.97~−1.73	AIS	*qTGW2* [18]
*qETN11*	GY, BL	RM6288	11	19.35	2.83~3.03	20.44~21.72	−2.02~−1.23	IAS	*qTN11-1* [8]
*qNSP6*	GL, GY	RM6818	6	59.92	3.92~3.83	24.59~20.33	4.66~192.07	AIS	*GN1a* [27]
*qSS1*	GY, BL	RM5410	1	151.42	4.00~2.70	21.48~13.20	−9.89~−4.66	AIS, IAS	*qSS1.1* [30]

^a^ Peak values in LOD profile that exceed the threshold value; ^b^ phenotypic variance explained by the QTL. ^c^ A positive value indicates that the presence of the IR24 allele leads to an increase in the trait. The four environments and BLUE values are denoted by GL: Guilin; GY: Guiyang: NC: Nanchang; NJ: Nanjing; and BL: BLUE.

## Data Availability

The original genotypic and phenotypic data are available in Supplementary Materials.

## Supplementary Materials

The following supporting documents can be downloaded at https://www.mdpi.com/article/10.3390/plants14010043/s1: Data S1: Genotypic and phenotypic data of RILs and reciprocal SSSLs. Table S1: QTLs identified by BIP in each environment and BLUE. Table S2: QTLs identified by the combined mapping method of MET. Table S3: QTLs identified by the CSL functionality in reciprocal SSSL populations in each environment and BLUE. Table S4: The validation by reciprocal SSSLs for the eight stable QTLs. Figure S1. Schematic representation of physical map, linkage map, and stable QTLs identified and validated in populations RIL, AIS, and IAS. Figure S2: Indication of stable and validated QTLs on genetic linkage map constructed from the RIL population.
